# Pilot study comparing operating room workflow and team ergonomics in robotic-assisted versus navigated total knee arthroplasty

**DOI:** 10.1051/sicotj/2026026

**Published:** 2026-05-05

**Authors:** Thomas Aubert, Willem Franssen, Aline Vandeputte

**Affiliations:** 1 Groupe Hospitalier Diaconesses Croix Saint Simon 125 Rue d’Avron Paris 75020 France; 2 DEO.Care Beringen Belgium

**Keywords:** Robotic-assisted TKA, Navigated TKA, Operating room time, Operating room workflow, Surgeon’s ergonomics

## Abstract

*Background*: Robotic-assisted systems have been developed to improve the accuracy and reproducibility of total knee arthroplasty (TKA). While outcomes have been widely studied, the effects of these systems on intraoperative workflow and surgical team workload have received less attention. The aim of this study was to compare procedural setup, efficiency, workload, and ergonomics between the VELYS robotic-assisted solution (VRAS) and computer-navigated TKA (NAVI). *Methods*: Twenty patients who underwent primary TKA performed by a single surgeon, using a single implant type, were enrolled in this research (10VRAS, 10NAVI). Procedural efficiency was assessed by reference to an AI-backed process digital twin platform. Workload was evaluated using NASA-TLX questionnaires, objective ergonomic measures (power tool holding times, retractor holding times, and leg holding times), and a tray analysis. *Results*: The mean total operating room (OR) time was 69.4 min for the VRAS group and 72.9 min for the NAVI group, with no significant difference. The preparation (22 min) and the breakdown times (12.6 vs.11.7 min) were equivalent. The skin-to-skin times averaged 34.3 min for the VRAS group versus 38.9 min for the NAVI group. NASA-TLX scores revealed significantly lower mental, physical, and temporal demands, reduced effort and frustration, and better perceived performance of the surgeon in the VRAS group (*p* < 0.05). The instrument burden was similar, 5 trays (21.5 kg) for VRAS and 4 trays (20.9 kg) for NAVI. The objective workload was reduced for the VRAS group, with shorter power tool holding (2.7 vs. 7.7 min, *p* < 0.001), retractor holding (7.8 vs. 13.0 min, *p* = 0.01), and leg holding times (3.4 vs. 4.7 min, *p* = 0.02). *Discussion*: Compared with navigated TKA, robotic assistance did not prolong overall OR time and was associated with lower measured NASA-TLX scores. These findings suggest that robotic-assisted TKA may offer workflow and ergonomic advantages, although further studies with larger samples are needed to confirm these preliminary observations. *Level of evidence*: Level 4, retrospective study.

## Introduction

Total knee arthroplasty (TKA) is a common procedure, and its incidence is expected to increase significantly in the near future [1]. While TKA generally provides good clinical outcomes, up to 20% of patients remain dissatisfied [2]. Considering the patient’s functional phenotype and using personalized implantation strategies could improve functional results [3], but this approach requires technological assistance that is capable of assessing intraoperative ligament balance, such as navigation systems.

More recently, the development of robotic-assisted TKA has the potential to increase surgical accuracy and allow for more precise execution of the preoperative plan [4]. While most related research has focused on patient outcomes, the effects of robotic-assisted TKA on the intraoperative workflow and surgical team workload remain underexplored. In addition to accuracy considerations, understanding how these technologies influence operating room (OR) processes has become increasingly relevant. Beyond the relatively short learning curve [5], robotics is associated with a longer phase before achieving stabilization and a reduction in operative time, raising concerns regarding overall procedure duration compared with conventional techniques.

Most studies comparing operative times have focused only on skin-to-skin duration, and few studies have investigated overall operating room (OR) occupancy [6]. No studies have comprehensively compared computer-navigated TKA (NAVI) and robotic-assisted TKA across the entire OR process, extending from patient entry to room breakdown. The adoption of new technologies also requires additional setup and installation, which may introduce workflow variations that are not yet fully quantified [7].

OR efficiency is a critical issue. As the healthcare system continues to transform, arthroplasty becomes predominant in the private sector in some countries [8], and the association between lower surgical volumes and decreased profit margins develops [9], the cost implications of robotic systems must be considered. Although robotic-assisted TKA is associated with higher acquisition costs, some studies suggest that these costs may be offset by reduced complication rates and shorter lengths of stay [10]. Nevertheless, optimization of the OR workflow remains essential [11]. New technologies must therefore avoid negatively impacting OR efficiency, particularly since the majority of hospital revenues are generated by the OR [12]. Reducing OR delays improves resource utilization, enhances staff efficiency, increases patient satisfaction, and decreases operating costs [13].

Ergonomic assessment has also become increasingly important, as surgical procedures often involve prolonged static postures, forceful instrument handling, and high cognitive demand [14]. Recent literature has highlighted that while robotic technologies improve implant accuracy, they may also affect surgeon ergonomics and workload, effects which remain underreported [15]. Another factor that should be considered is the number and weight of trays and instruments needed, some of which may not be used but still necessitate handling, sterilization, and storage [12].

Finally, surgeon and staff satisfaction is important. Evidence suggests that OR staffing and the surgical environment significantly influence patient outcomes, although the well-being of healthcare professionals remains underrepresented in current optimization strategies [16]. In this context, evaluating both workflow and team-related parameters provides a more comprehensive assessment of how new technologies are integrated into routine practice.

The objectives of this study were to analyze the impact of robotic-assisted TKA using the VELYS Robotic-Assisted Solution (VRAS) versus NAVI on operating room efficiency, the number and weight of instrument trays, and surgical team workload and ergonomics. Given the limited sample size, this investigation was designed as an exploratory pilot study aimed at identifying potential workflow and ergonomic differences rather than providing definitive comparative conclusions.

## Patients and methods

### Patients

A total of 20 patients met the inclusion criteria: 10 underwent VELYS Robotic-Assisted Solution TKA (VRAS), and 10 underwent Knee3, Brainlab navigated TKA (NAVI) between January and May 2025. Patients were included consecutively. No randomization was performed. The surgical technique was not determined by patient characteristics but depended on operating room scheduling and system availability.

All procedures were performed by one surgeon, experienced in both techniques, with a similar operating room set-up. The surgeon had several years of prior experience with navigated TKA, and robotic-assisted TKA was performed only after completion of the initial learning phase [17]. A cemented cruciate-retaining prosthesis without patellar resurfacing was implanted according to an inverse kinematic alignment philosophy (Attune, Johnson and Johnson Medtech). The inclusion criteria were adult men and women with end-stage osteoarthritis (Kellgren–Lawrence grade III or IV) who underwent primary TKA. The only exclusion criteria were revision TKA and unicompartmental knee arthroplasty (UKA). No exclusion criterion was applied regarding the degree of preoperative varus or valgus deformity in order to reflect routine clinical practice. This study was approved by the local institutional review board (IRB 01 2025_25Orth).

### Data Collection

The operational excellence of the procedures and team ergonomics were monitored using both the AI-enabled camera application of an independent company (DEO NV, Belgium) and an experienced medical engineer attending the procedures in real time. For each procedure, a fixed camera was systematically positioned in the same corner of the operating room. This position allowed simultaneous visualization of the operating table, instrument tables, and the entire surgical staff. The standardized recording conditions ensured comparable ergonomic analysis between both techniques. A total of 108 VRAS-specific timestamps and 88 NAVI-specific timestamps were captured per case via the DEO AI-backed process digital twin platform.

The total OR time in this study was calculated as the interval from the start of the first activity (patient's entrance into the OR) until the end of the last activity (patient's exit from the OR). For the procedures under investigation, the first activity was the patient entering the OR. The last activity occurred when the patient left the OR.

**Figure 1:**
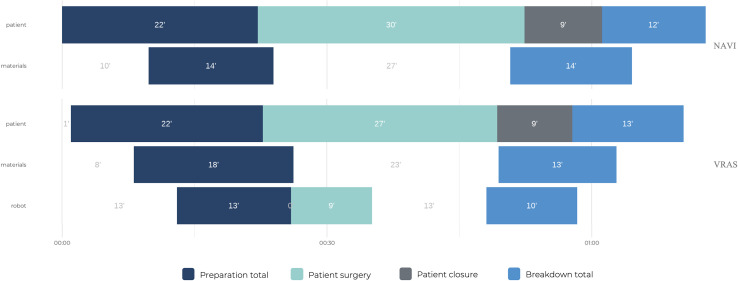
Definitions.

For each procedure, three major phases and seven subphases were differentiated (Figure 1):

1. **Preparation:** The interval from patient entry into the OR until incision, including the preparation of the patient, the instruments, and the technology system.
2. **Skin-to-skin (surgery + closure):** The interval from incision until the completion of wound closure. The skin-to-skin interval is further subdivided into the following intervals: Knee preparation (knee approach, landmarking, evaluation, and surgical planning), resection, implant trial, and implant insertion.
3. **Breakdown:** The interval from the completion of wound closure until the last activity (patient leaving OR).

The OR team responsible for both robotic-assisted and NAVI TKA consisted of 5 people, including an experienced surgeon, a fellow, an instrumenting nurse, 1 circulating nurse and an anesthesia nurse. All procedures were performed in the same operating room using an identical environmental configuration. The surgical team composition, staff positioning, and number of personnel were strictly standardized for all procedures.

Data concerning staff OR presence, tasks, and responsibilities were collected by annotating the stakeholders present in the digital twin tool, linking staff to tasks, and collecting sterile and nonsterile times. Additional collected data on OR team well-being and ergonomics included NASA-TLX questionnaires, the number and weight of instrument trays used, power tool holding time, retractor holding time, leg holding time, and recut frequency. The NASA-TLX questionnaires were completed by 2 staff members: the surgeon and the instrumenting nurse.

### Statistical analysis

Continuous variables, including time metric data, NASA-TLX scores, body mass index (BMI), age, and coronal alignment parameters, were described using means, standard deviations (SDs), ranges, medians, and interquartile ranges (IQRs). Categorical variables, including gender, ASA classification, and operation side, are summarized as numbers and percentages. No formal sample size calculation was performed, as this study was designed as an exploratory pilot study including all consecutive eligible cases during the study period.

The Shapiro–Wilk test was used to assess the normality of continuous variables. Normally distributed continuous variables were compared using the independent *t*-test. Equality of variances was assumed using Levene’s test for equality of variances. Non-normally distributed continuous variables were analyzed using the Mann–Whitney *U* test. Effect sizes for differences in continuous variables were reported as the mean differences (MDs) along with their 95% confidence intervals (CIs). Categorical variables were compared using the chi-square test of independence. If the expected frequency in the contingency table was less than 5 in more than 20% of the cells, Fisher’s exact test was used. Statistical significance was set at a *p*-value of <0.05. All the statistical analyses were performed with the assistance of SPSS software (V29.2.0).

## Results

The baseline demographic characteristics of the participants, comparable between the two groups, are reported in Table 1.

**Table 1:** NAVI and VRAS cohort characteristics.

	NAVI				VRAS				Effect size		
Characteristics	Mean ± SD *n* (%)	Range	Median	IQR	Mean ± SD *n* (%)	Range	Median	IQR	*p*-value	MD	95% CI
Age (years)	71.10 ± 10.68	45.00–84.00	73.00	69.25–76.50	70.80 ± 5.94	63.00–83.00	70.50	67.50–72.50	n.s.	0.3	−7.8 to 8.4
BMI (kg/m^2^)	29.17 ± 4.59	23.80–36.00	28.10	25.45–32.13	28.39 ± 4.18	22.70–35.00	28.40	25.85–30.98	n.s.	0.78	−3.3 to 5
LDFA (deg.)	90.70 ± 2.79	86.00–94.00	91.50	88.25–92.75	89.20 ± 3.65	84.00–95.00	89.00	87.25–90.00	n.s.	1.5	−1.6 to 4.6
mHKA (deg.)	179.90 ± 6.35	171.00–190.00	178.00	176.50–184.25	182.00 ± 13.15	166.00–205.00	181.50	171.25–189.50	n.s.	−2.1	−12.1 to 7.9
MPTA (deg.)	88.40 ± 2.67	84.00–92.00	88.50	88.00–90.00	88.00 ± 2.67	84.00–93.00	88.00	86.25–89.75	n.s.	.40	−2.1 to 2.9
aHKA (deg.)	−2.30 ± 3.59	−8.00– 5.00	−2.50	−3.75 to −1.25	−1.20 ± 6.12	−11.00–9.00	−1.50	−4.25–2.50	n.s.	1.1	−3.38 to 5.58
Gender**											
Women	6 (60%)				8 (80%)				n.s.		
Men	4 (40%)				20 (20%)						
Side**											
Left	7 (70%)				4 (40%)				n.s.		
Right	3 (30%)				6 (60%)						
ASA Classification											
I	1 (10%)				0 (0%)				n.s.		
II	7 (70%)				4 (40%)						
III	2 (40%)				6 (60%)						

*Nonparametric value based on the Mann–Whitney *U* test. **Categorical value based on Fisher’s exact test.

Abbreviations: Navi: navigation; VRAS: Velys robotic-assisted surgery; ASA: American Society of Anesthesiologists; deg.: degrees; BMI: body mass index; SD: standard deviation; IQR: interquartile range; MD: mean difference; CI: confidence interval; MPTA: medial proximal tibial angle; mHKA: mechanical hip–knee angle; aHKA: arithmetic hip–knee angle; LDFA: lateral distal femoral angle.

### OR efficiency analysis

**Figure 2:**
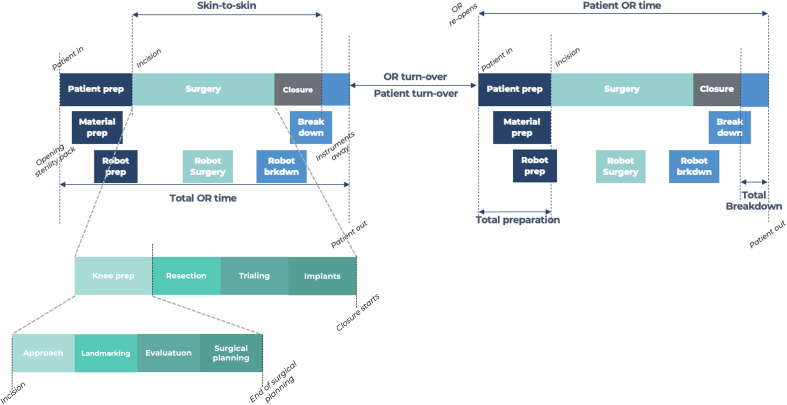
Comparisons of total OR time and subdivisional flow times between the NAVI (*n* = 10) and VRAS (*n* = 10) groups.

The time analysis results are described in Table 2 and Figure 2. No significant differences were observed between the groups for total OR time (MD 3.47 min, 95% CI −3.0–9.9; *p* > 0.05), preparation time (MD 0.39, 95% CI −2.4–3.2; *p* > 0.05.), surgery time (MD 4.59 min, 95% CI −0.1–9.2; *p* > 0.05), or breakdown time (MD −0.89 min, 95% CI −0.4–4.6; *p* = n.s.). Additionally, with respect to the surgical subdivisional flow times, no significant differences were observed, except for TKR resection (MD 3.06, 95% CI 1.5–5.0; *p* = 0.03), which resulted in faster tibial and femoral resection in the VRAS group. A post hoc power analysis was performed based on the observed effect sizes. The achieved statistical power was generally low (<30%) for most variables, except for TKR resection time, which showed a large effect size (Cohen’s d ≈ 0.9; power ≈ 50%). Further research with a larger sample size or additional controls may be warranted to explore this potential difference.

**Table 2:** NAVI and VRAS time analysis results.

	NAVI				VRAS				Effect size		
Characteristics	Mean ± SD	Range	Median	IQR	Mean ± SD	Range	Median	IQR	*p*-value	MD	95% CI
Total OR time (min)	72.87 ± 6.72	61.37–80.95	74.49	67.48–78.36	69.40 ± 7.08	60.07–83.32	67.86	65.81–70.58	n.s.	3.47	−3.0 to 9.9
Preparation time (min)	22.16 ± 3.39	15.48–27.02	21.33	20.81–24.27	21.77 ± 2.52	16.23–25.77	21.76	21.10–22.92	n.s.	0.39	−2.4 to 3.2
Surgery time (min)	38.89 ± 4.39	32.73–45.60	38.09	35.43–42.70	34.30 ± 5.43	26.98–43.75	33.58	30.19–36.38	n.s.	4.59	−0.1 to 9.2
Breakdown time (min)*	11.71 ± 2.94	8.58–19.32	11.08	10.20–11.87	12.61 ± 2.98	8.92–18.78	11.85	10.95–14.33	n.s.	−.89	0.4 to 4.6
Prep knee (min)	9.87 ± 1.42	8.32–12.67	9.28	8.85–10.94	10.66 ± 1.39	8.83–12.92	10.30	9.70–11.63	n.s.	−.79	−2.1 to 0.5
TKR resection (min)	11.63 ± 2.21	9.07–16.02	11.43	10.03–11.87	8.57 ± 1.79	5.47–10.98	8.62	7.69–9.61	0.03	3.06	1.2 to 5.0
TKR trial (min)	2.75 ± 1.29	1.07–4.55	2.61	1.82–3.90	1.95 ± 0.93	0.65–3.80	1.76	1.55–2.50	n.s.	.80	−0.7 to 5.2
TKR implants (min)	5.97 ± 1.61	3.77–8.33	5.68	4.87–7.22	5.34 ± 0.98	3.23–6.50	5.44	5.13–6.00	n.s.	.63	−0.3 to 1.9
Sewing* (min)	8.67 ± 2.72	6.37–14.30	7.78	6.89–8.59	7.79 ± 3.55	4.53–12.45	5.88	4.78–11.59	n.s.	.88	−2.1 to 3.9
Knee approach (min)	6.60 ± 0.58	5.88–7.90	6.68	6.19–6.79	7.28 ± 1.38	5.13–9.70	7.27	6.44–7.70	n.s.	−.68	−1.7 to 0.3
Landmarking (min)*	1.52 ± 0.54	1.08–2.85	1.38	1.13–1.54	1.74 ± 0.31	1.25–2.27	1.73	1.53–1.94	n.s.	−.23	−0.6 to 0.2
Evaluation (min)	0.58 ± 0.27	0.28–1.22	0.53	0.40–0.64	0.73 ± 0.37	0.25–1.45	0.57	0.52–0.94	n.s.	−.15	−0.5 to 0.2
Surgical planning* (min)	1.18 ± 0.69	0.60–2.48	0.87	0.70–1.53	0.91 ± 0.40	0.35–1.52	0.93	0.60–1.23	n.s.	.26	−0.3 to 0.8

*Nonparametric value based on the Mann–Whitney *U* test.

Abbreviations: Navi: navigation; VRAS: Velys robotic-assisted surgery; SD: standard deviation; IQR: interquartile range; MD: mean difference; CI: confidence interval.

**Figure 3:**
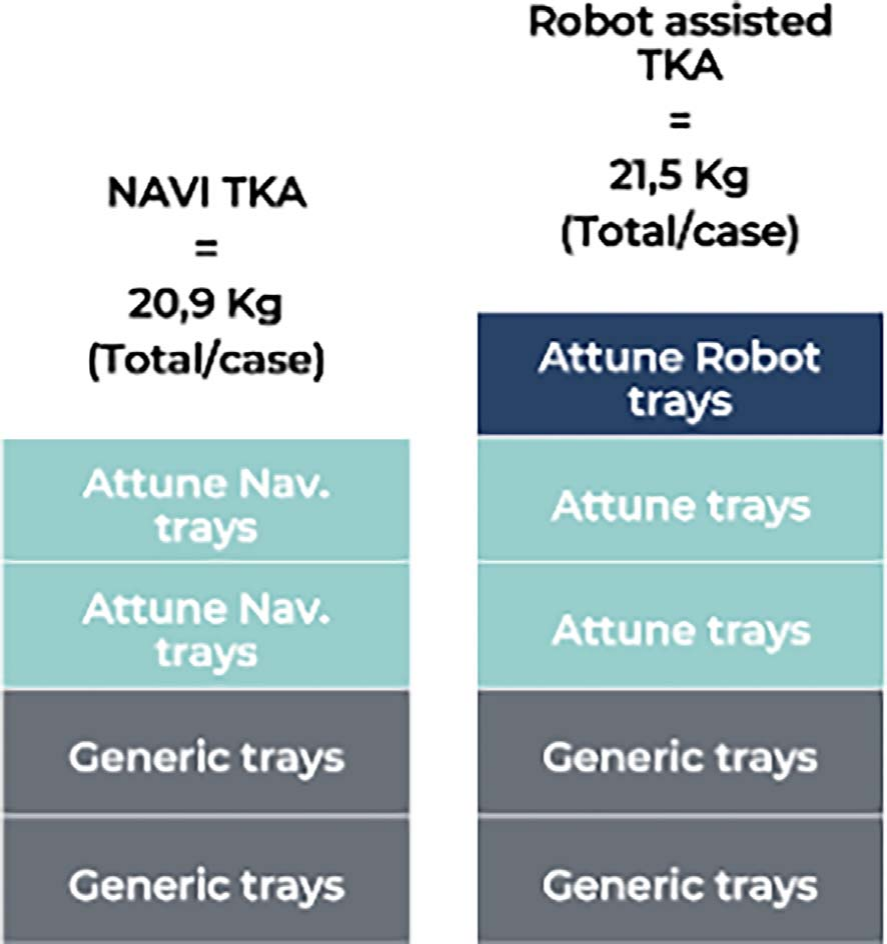
Instrumentation setup.

The optimized instrumentation setup was not statistically different between the VRAS and NAVI groups, consisting of 5 trays (21.5 kg) and 4 trays (20.9 kg), respectively (Figure 3).

### OR team well-being and ergonomics analysis

The results of the analysis of the ergonomic data are described in Tables 3 and 4. Significant differences were observed for all six NASA dimensions for the surgeon (*p* < 0.001). No significant differences were observed for the scrub nurses (*p* > 0.05). Other ergonomics parameters included significant differences between the NAVI and VRAS groups for power tool holding time (MD 4.9, 95% CI 3.6–6.3; *p* = <0.001), retractor holding time (MD 5.2, 95% CI 1.6–8.9; *p* = 0.01) and leg holding time (MD 1.4, 95% CI 0.3–2.5; *p* = 0.02), indicating reduced mental and physical fatigue for the OR team during VRAS procedures. Recut frequency did not differ between the groups.

**Table 3:** NAVI and VRAS NASA-TLX questionnaire results.

	NAVI			VRAS			
Characteristics	Mean ± SD	Range	Median	Mean ± SD	Range	Median	*p*-value
Surgeon (NAVI *n* = 10 and VRAS *n* = 9)							
Mental demand	7.20 ± 2.94	4.00–14.00	6.50	2.78 ± 0.97	1.00–4.00	3.00	<.001
Physical demand*	4.50 ± 1.18	3.00–7.00	4.00	2.44 ± 1.33	1.00–5.00	2.00	<.001
Temporal demand	6.40 ± 1.78	4.00–10.00	6.00	3.33 ± 1.50	1.00–6.00	3.00	<.001
Performance*	6.80 ± 2.39	4.00–11.00	6.00	2.22 ± 0.67	1.00–3.00	2.00	<.001
Effort*	6.50 ± 1.90	4.00–9.00	6.00	3.11 ± 1.17	2.00–5.00	3.00	<.001
Frustration*	8.20 ± 3.68	5.00–14.00	6.00	2.67 ± 1.22	1.00–4.00	3.00	<.001
Scrub nurse (NAVI *n* = 10 and VRAS *n* = 8)							
Mental demand*	6.70 ± 3.16	3.00–13.00	6.50	5.88 ± 2.23	4.00–11.00	5.50	n.s.
Physical demand*	6.10 ± 2.64	4.00–13.00	6.00	6.63 ± 2.56	4.00–11.00	7.00	n.s.
Temporal demand*	7.90 ± 2.23	6.00–13.00	7.50	9.50 ± 4.44	5.00–16.00	7.50	n.s.
Performance*	4.80 ± 1.69	2.00–7.00	4.50	6.00 ± 3.07	4.00–12.00	4.00	n.s.
Effort	8.10 ± 2.60	5.00–12.00	8.00	7.63 ± 4.24	4.00–16.00	6.50	n.s.
Frustration	4.10 ± 2.73	1.00–10.00	4.00	7.75 ± 5.31	1.00–17.00	7.00	n.s.

*Nonparametric value based on the Mann–Whitney *U* test.

Abbreviations: Navi: navigation; VRAS: Velys robotic-assisted surgery; SD: standard deviation; TLX: task load index.

**Table 4:** Other NAVI and VRAS ergonomic factors.

	NAVI				VRAS				Effect size		
Characteristics	Mean ± SD	Range	Median	IQR	Mean ± SD	Range	Median	IQR	*p*-value	MD	95% CI
Power tool holding (min)	7.66 ± 1.90	5.43–11.07	6.97	6.29–9.13	2.69 ± 0.68	1.67–3.57	2.67	2.28–3.23	<.001	4.96	3.6 to 6.3
Retractor holding (min)	13.03 ± 5.06	7.40–23.75	11.38	9.73–15.93	7.80 ± 1.19	5.28–9.48	7.72	7.37–8.30	0.01	5.23	1.6 to 8.9
Leg holding (min)	4.70 ± 1.48	3.12–7.45	4.68	3.31–5.45	3.35 ± 0.80	2.25–4.25	3.53	2.60–4.03	0.02	1.35	0.3 to 2.5
Trays											
Number of trays	4				5				/		
Tray weight (kg)	21.5				20.9				/		
Recuts*											
Tibial or femoral	2 (20%)				2 (20%)				n.s.		

*Categorical value based on Fisher’s exact test.

## Discussion

The emergence of new technologies may improve functional outcomes, but their integration into the operating room should not result in delays or reduced efficiency [11, 18]. In this pilot comparison of robotic-assisted and computer-navigated TKA, no statistically significant differences were observed in overall operative time between techniques. Although the VRAS group showed a numerically shorter average total OR time (69.4 min for VRAS TKA versus 72.9 min for NAVI), this difference should be interpreted cautiously, given the limited sample size. The number of instrument trays was similar, and only a negligible difference in weight (0.6 kg) as observed. In contrast, significant differences were observed in the ergonomic assessment. All six NASA-TLX domains improved for the surgeon in the VRAS procedures (*p* < 0.001), whereas no differences were detected for the scrub nurses. Objective ergonomic parameters, including power tool holding time, retractor holding time, and leg holding time, were also significantly reduced in VRAS procedures, suggesting a potential reduction in physical and cognitive strain for the operating surgeon.

The absence of significant differences in operative time between the two techniques, with a trend toward shorter times with robotic-assisted procedures, aligns with the current literature [6]. The main difference, although not statistically significant, was observed in skin-to-skin time (34.3 vs. 38.9 min), largely explained by faster bone-cut execution in the robotic procedures (an average gain of 3 min) related to the absence of mechanical guide positioning and iterative adjustments required in navigated techniques. Peripheral times, including preparation, OR occupation, and breakdown, were comparable. Although some studies have reported increased operative times due to the use of a robotic setup [19], other evidence suggests that once the learning curve stabilizes, robotic procedures may achieve similar or shorter durations compared with conventional techniques [6, 17, 20]. However, most previous studies have focused solely on skin-to-skin duration and not analyzed the entire OR workflow. In our workflow, preparation steps were intentionally organized in parallel. While the scrub nurse performed system start-up and saw blade calibration, the surgeon performed surgical hand antisepsis. After draping, robotic arm calibration was completed simultaneously with the surgical exposure. This standardized team organization likely contributed to limiting the additional time commonly attributed to robotic setup. The present study contributes additional descriptive data by evaluating the full sequence from room entry to room breakdown and indicates that robotic integration did not adversely affect global OR processes in this early experience.

The difference in weight between the two instrumentation systems was only 0.6 kg. The operating room setting is associated with unique occupational hazards and extremely high ergonomic demands, particularly related to heavy equipment and surgical supplies. Injuries among perioperative nurses are increasing [21], and working full-time as a scrub nurse significantly increases the risk of upper limb work-related musculoskeletal disorders (WMSDs) [21]. At-risk safety behaviors among perioperative nursing teams remain a concern, and current guidelines recommend a maximum weight of 11 kg per surgical instrument set [22]. Given that both systems used in our study involved a similar number of trays, whose weights were below these recommendations, robotic-assisted surgery does not appear to expose paramedical staff to additional ergonomic risk.

Our findings demonstrate that VRAS significantly reduces both subjective and objective ergonomic burdens on surgeons. Improved NASA-TLX scores across all six domains, combined with reduced tool, retractor, and leg holding times, indicate that VRAS procedures are associated with lower mental demand, physical effort, and frustration. This difference may be related not to planning itself, which is provided by both technologies, but to execution control, as robotic assistance provides controlled bone resection and reduces the need for continuous manual verification. WMSDs are a substantial concern for orthopedic surgeons, with a systematic review reporting a lifetime prevalence rate of 37–97% [14]. This recent study urged institutions to include surgeon ergonomics in the routine evaluation of new surgical technologies. The incorporation of workload monitoring into practice, including attention to equipment positioning, adjustment, and postural analysis, could help mitigate the occurrence of WMSDs [23]. A study comparing conventional TKA with robotic-assisted TKA demonstrated that robotic-assisted TKA resulted in decreased physiologic stress on the surgeon, lower energy expenditure per minute, and reduced postural strain [24], similar to the use of patient-specific implants [25]. The ergonomic improvements observed in the current study support the hypothesis that robotic assistance may positively influence surgeon well-being, although these findings require confirmation in larger cohorts. Beyond comfort considerations, the surgeon’s well-being represents an important implication of ergonomic optimization. Increased physical and cognitive workload is associated with fatigue and reduced concentration during surgery. Therefore, improving ergonomics may indirectly contribute to surgical safety while also supporting long-term occupational health and career sustainability in orthopedic surgery [26].

Several strengths of this study should be noted. First, all procedures were performed by the same experienced surgeon, thus ensuring the consistency of the surgical technique and eliminating potential bias related to the learning curve when robotic-assisted TKA was introduced. Data collection was also enhanced through the use of video recordings of OR sessions, thereby reducing the risk of observation bias compared with direct monitoring by an external observer.

Despite these strengths, several limitations must be acknowledged. Most importantly, the sample sizes of the VRAS and NAVI groups were relatively small, which may limit the generalizability of this research and affect the robustness of the statistical analysis. Our post hoc power analysis indicates that the study was underpowered to detect small-to-moderate differences. In addition, group allocation was not randomized due to the retrospective study design. Accordingly, the non-significant differences in operative time should not be interpreted as equivalence between techniques. Nevertheless, the sample sizes were sufficient to demonstrate statistically significant differences in ergonomic and workload outcomes between groups, although the risk of type I error related to multiple comparisons cannot be excluded. With the exception of TKA resection time, no significant differences were observed between groups. Importantly, the non-significant trend towards shorter operative times with VRAS does not contradict our main conclusion. Moreover, while a single-surgeon design ensures procedural consistency, it may also limit external validity, as the results may not be fully representative across other surgeons or institutions. The reproducibility of the operative time, preparation, and breakdown across different teams should ideally be assessed in future multicenter studies. Finally, the study was limited to the analysis of OR-related variables; no clinical or radiological outcomes were examined. However, previous reports have suggested that the operative duration may be correlated with functional results, in which context shorter operative times have been associated with improved post-TKA Forgotten Joint Scores [27]. Future studies combining workflow analysis with clinical endpoints would therefore be valuable to better understand the implications of OR efficiency on postoperative recovery.

## Conclusion

Compared with navigated TKA, the implementation of robotic-assisted technology in TKA did not demonstrate any detrimental effect on overall OR efficiency and was associated with lower measured NASA-TLX scores and reduced operative task durations. These findings should be interpreted within the exploratory nature of this pilot study, but suggest that robotic systems may indicate differences in measured ergonomic and workload parameters without prolonging the procedure. Further studies with larger cohorts and multicenter designs are needed to confirm these preliminary observations and to better define their relevance within value-based care models.

## Data Availability

The data that support the findings of this study are available from the corresponding author upon reasonable request.
